# Effects of bFGF on the Modulation of Apoptosis in Gingival Fibroblasts with Different Host Ages

**DOI:** 10.1155/2013/619580

**Published:** 2013-09-10

**Authors:** Kotaro Tanimoto, Satoru Ohkuma, Yuki Tanne, Ryo Kunimatsu, Naoto Hirose, Tomomi Mitsuyoshi, Yuki Yoshimi, Shaoching Su, Kazuo Tanne

**Affiliations:** Department of Orthodontics, Applied Life Sciences, Hiroshima University Institute of Biomedical & Health Sciences, 1-2-3 Kasumi, Minami-ku, Hiroshima 734-8553, Japan

## Abstract

The purpose of this study was to investigate the effects of basic fibroblast growth factor (bFGF) treatment on the proliferation and apoptosis of cultured gingival fibroblasts (GFs). Human GFs were isolated from the palatal gingival tissues of 16 healthy volunteers ranging in the age from 9 to 35 years old. Cultured GFs were subjected to the analyses for cell proliferation by ELISA assay, gene expression by RT-PCR analysis, and apoptosis potency by caspase-3 assay. The cell proliferation activity and gene expression of type-I collagen and caspase-3 activity were enhanced significantly by the treatment with bFGF in cultured GFs. Furthermore, the activity of caspase-3 in cultured GFs from young subjects was significantly higher than that in GFs from adults. It is shown that bFGF significantly enhances the gene expression of type-I collagen in cultured fibroblasts from human gingival tissues. It also demonstrated that bFGF modulates the apoptosis of periodontal fibroblasts, and the effect is higher in young subjects, indicating a significant role of bFGF in the prevention of scar formation during wound healing.

## 1. Introduction

Basic fibroblast growth factor (bFGF) is a multigene family member of structurally related peptide growth factors, and its function is mediated through high-affinity receptors [1]. It is well known that bFGF is a multifunctional cytokine to participate in the process of wound healing, cell proliferation, and apoptosis [2–4].

Wound healing can be divided into three consecutive, partly overlapping phases: inflammation, proliferation, and tissue remodeling [5]. The macrophage plays a pivotal role in the transition between wound inflammation and repair, since this cell both scavenges tissue debris and releases a plethora of biologically active substances that include growth factors like bFGF. During the remodeling phase, the number of blood vessels declines and apoptosis of fibroblasts results in scar tissue with a low cell density [6].

Apoptosis is a requisite event for maintaining kinetic homeostasis in continuously renewing tissues such as oral mucosa and skin, and it is suggested to play a crucial role in the repair of connective tissues. Nevertheless, apoptosis is often involved in pathogenetic pathways [7]. Regarding the mechanism of apoptosis induced by bFGF treatment, it has recently been demonstrated that bFGF plays an important role in apoptosis during development of the neural retina. The apoptosis of fibroblasts treated with bFGF was enhanced in both *in vivo* and *in vitro* experiments [8, 9]. In dentistry, bFGF was reported to enhance the proliferation of human periodontal ligament (PDL) cells in beagle dogs [10, 11]. However, the mechanisms of apoptosis enhanced by bFGF, and the age difference still remains unclear.

The purpose of this study was to investigate the effects of bFGF treatment on the metabolism of cultured GFs from different-aged hosts with a special reference to the expressions of caspase-3. These results support our hypothesis that the temporal activation of bFGF at the injury site results in effective apoptosis of granulation tissue fibroblasts, a process that is the initiation of wound remodeling phases.

## 2. Materials and Methods

### 2.1. Surgical Procedure of Scar Formation and bFGF Injection

In this experiment, 40-male-Wistar rats aged 6 and 12 weeks were used. The rats were equally divided into two groups: a bFGF injection group and a control group. The protocol was approved by the Animal Care and Use Committee of Hiroshima University. In the scar formation, rectangular strips of the bilateral one third of the hard palatal mucoperiosteum were excised under general anesthesia induced by intraperitoneal injection of sodium pentobarbital (1 mg/kg of Nembutal, Dainabot, Osaka, Japan). The exposed bone surface was wiped with a cotton pellet for complete removal of the periosteum. Five days after excision, 10 *μ*L of bFGF solution (20 mg of bFGF/10 *μ*L saline) and 10 *μ*L of PBS were injected into the operated area, respectively.

### 2.2. Histological Preparation

At 10 days after the excision, the head was resected under deep anesthesia with diethyl ether and fixed by immersion. The specimens were decalcified in 22.5% ethylenediaminetetraacetate (EDTA) and embedded in paraffin, and serial frontal sections of 7 *μ*m in thickness were prepared, stained with ApopTag Plus Peroxidase In Situ Apoptosis Detection Kit (Millipore, Billerica, MA), and observed by light microscopy.

### 2.3. Cell Isolation and Culture

Permission for a series of experiments in this study was granted by the Ethics Committee of Hiroshima University. Human GFs were isolated from healthy palatal gingival tissues obtained from 16 patients before and during orthodontic treatment ranging in the age from 9 to 35 years old according to the methods described previously [12]. 16 entries were divided into the young group from 9 to 12 years old (*n* = 8; 2 male and 6 female) and the adult group from 26 to 35 years old (*n* = 8; 1 male and 7 female). Informed consent was obtained from all the patients prior to the beginning of experiments.

The explants were cultured in 100 mm dishes (Corning, New York, NY) with 10 mL Dulbecco's modified Eagle medium (DMEM; Nissui Pharmaceutical Co., Tokyo, Japan) containing 10% FBS (Mitsubishi-Kasei, Tokyo, Japan), 32 U/mL penicillin-G (Sigma, St. Louis, MO), 60 *μ*g/mL kanamycin (Meiji-Seika, Tokyo, Japan), and 250 ng/mL amphotericin B (ICN Biomedicals Inc., Aurora, OH), under an atmosphere of 5% CO_2_ in a humidified incubator at 37°C. The medium was changed every other day until the cells became confluent. The 5th–8th passaged cells isolated from 16 different gingival tissues were used for the following experiments. All results were obtained from the GF of all 16 individuals.

### 2.4. Cell Proliferation Assay

Proliferation of GFs was evaluated using a cell proliferation ELISA BrdU Kit (Roche Diagnostics, Basel, Switzerland). Briefly, GFs were trypsinized, seeded at a density of 1.0 × 10^3^ cells/well in 125 *μ*L DMEM on a type-I collagen (10 *μ*g/mL) coated 96-well plate (Falcon, Flanklin Lakes, NJ), and incubated overnight. GFs were washed three times with PBS and then incubated with or without bFGF (0.5, 1.0 ng/mL) (Peprotech, Paris, France) in the culture medium with 1% FBS. The culture medium of GFs with 1% FBS was used as negative control. GFs were incubated for 3, 5, 7, and 9 days. GFs were washed three times with PBS and 10 *μ*L of 5 *μ*g/mL 5-bromo-2′-deoxyuridine was added to each well and then incubated for 2 hr. After the incubation, optical density was measured at a wavelength of 570 nm by a Bio-Rad microplate reader (Model 550, Bio-Rad, Hercules, CA). GFs cultured in incomplete medium without bFGF were used as negative controls, and GFs treated with bFGF alone in incomplete medium were used as positive controls for each experiment. After 72 hrs, ELISA assay was performed, and the proliferation of GFs was compared by the percentage relative to negative controls.

### 2.5. Quantitative Real-Time PCR Analysis

The mRNA levels of fibronectin-1 and type-I collagen were examined by a quantitative real-time PCR analysis using a LightCycler system (Roche Diagnosics) and QuantiTectTM SYBR Green PCR Master Mix (Qiagen, Tokyo, Japan). GFs were seeded at a density of 6 × 10^4^ cells/well on 6-well plates (Falcon). The medium was changed every other day until 80% confluence. As the cells became 80% confluent, GFs were incubated with bFGF (Peprotech) at 0.5 or 1.0 ng/mL for 24 hrs and then washed excessively with PBS. Total RNA was extracted from the cell cultures using Trizol Reagent (Gibco BRL, Gaithersburg, MD). First-strand cDNA was synthesized from 1 *μ*g total RNA using Rever Tra Ace-*α* (Toyobo, Osaka, Japan).  Table 1 shows the sequences of the primers. The signals of fibronectin-1 and type-I collagens were evaluated in a qualitative manner, relative to the glyceraldehyde 3-phosphate dehydrogenase (GAPDH) signals. Normalized Ct values were expressed relative to the controls.

### 2.6. Caspase-3 Assay

GFs were seeded at a density of 1 × 10^3^ cells/well on 96-well plates (Falcon). The medium was changed every other day until 70% or 100% confluence. As the cells became 70% or 100% confluent, GFs were incubated with bFGF (Peprotech) at 0.5 or 1.0 ng/mL for 6–24 hrs and then washed excessively with PBS. After washing with PBS, PBS including NucView 488 Caspase-3 substrate (Biotium, SanFrancisco, CA) of 20 *μ*L was added to each well and incubated at 37°C for 1 hr. Caspase-3 activation was determined by fluorescence measurement using a fluorescence plate reader (model: ARVO SX; PerkinElmer, Yokohama, Japan) at a wavelength of 520 nm.

### 2.7. Statistical Analysis

The experiments were repeated at least in triplicate. Means and standard deviations were calculated from the data obtained, and differences in the means were examined by the use of a Student's *t-*test or one-way analysis of variance (ANOVA) followed by a Scheffe's multiple comparisons test at a significant level of *P* < 0.05 or *P* < 0.01. 

## 3. Results

**Figure 1:**
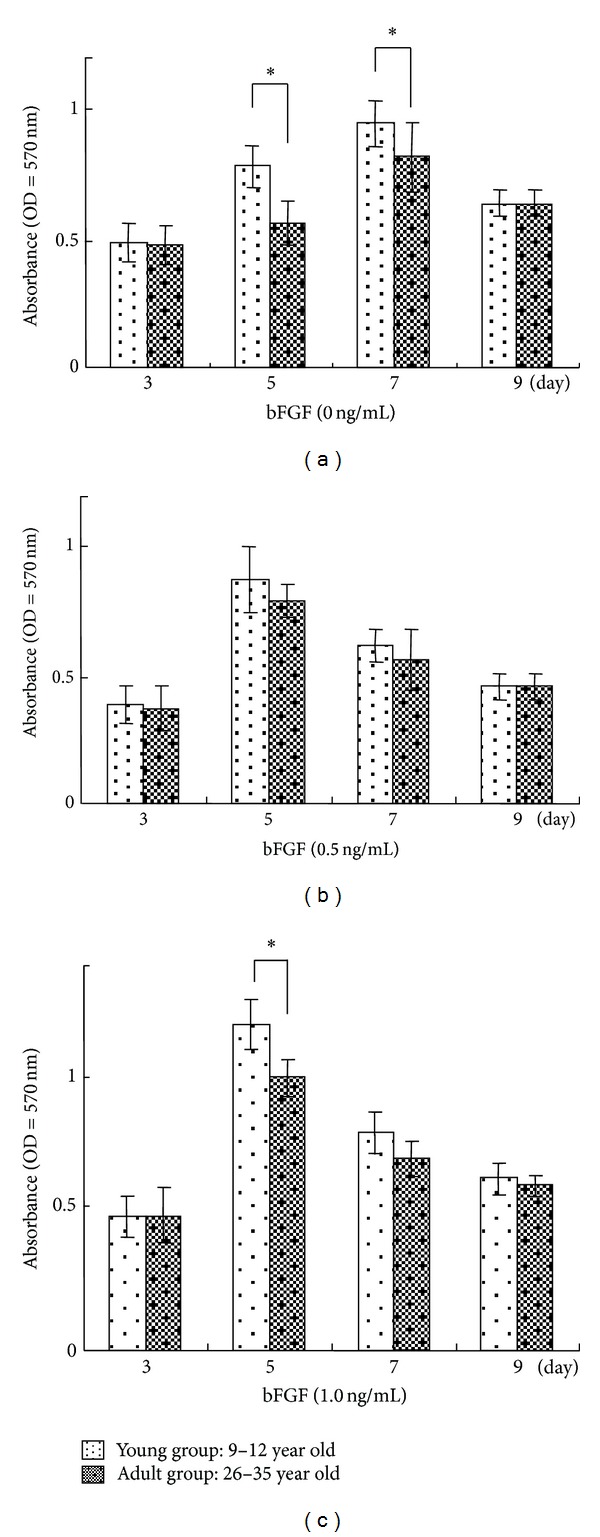
Proliferation of cultured human GFs induced by addition of bFGF. GFs were seeded at a density of 1.0 × 10^3^ cells/well. Cell proliferation of cultured GFs with 0 (a), 0.5 (b), and 1.0 (c) ng/mL bFGF was evaluated by measuring BrdU incorporated into the DNA of proliferating cells 3, 5, 7, and 9 days after seeding. Error bars indicate a standard deviation. *N* = 8,  *P < 0.05,  **P < 0.01.

In the young group, a significantly higher proliferation activity was shown when compared to the adult group on days 5 and 7 (Figure 1(a)). Meanwhile, the days that reached the peak were shortened on day 5 from day 7 by the treatment of bFGF (Figures 1(b) and 1(c)). After 1.0 ng/mL bFGF treatment, a significant difference in cell proliferation between young and adult groups was shown on day 5 (Figure 1(c)).

**Figure 2:**
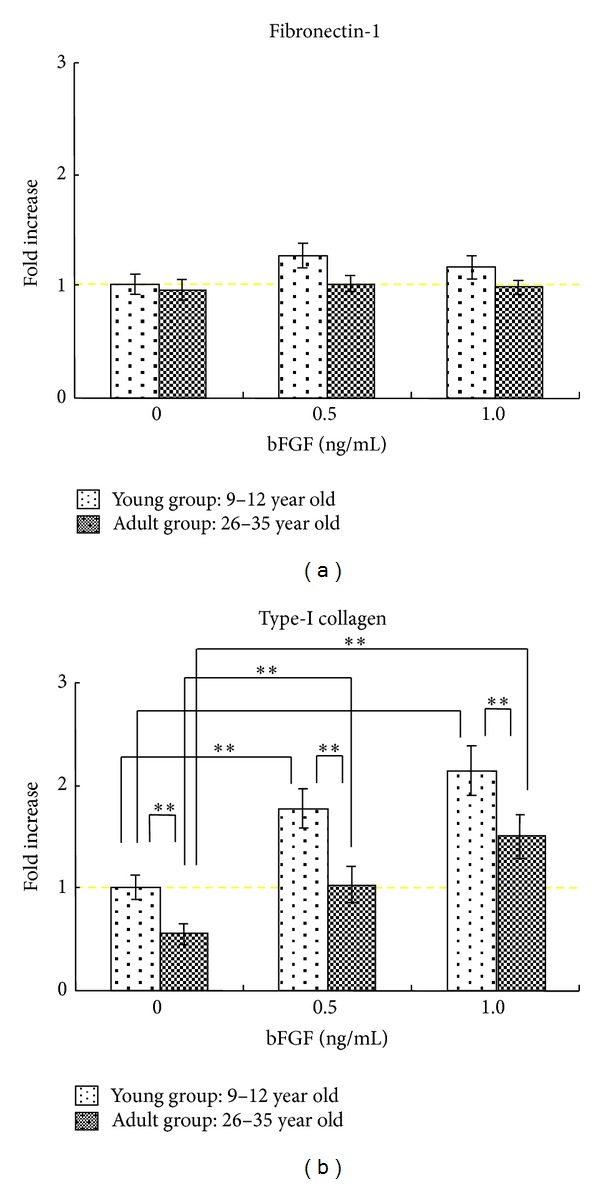
Changes in the gene expression of fibronectin-1 and type-I collagen during differentiation in cultured GFs. Total RNA was extracted from GFs as the cells became confluent; GFs were incubated with bFGF of 0.5 or 1.0 ng/mL for 24 hrs. The mRNA expression levels of fibronectin-1 (a) and type-I collagen (b) were determined by means of a real-time PCR analysis, normalized relative to the expression of GAPDH, and depicted as the rate of change in gene expression. Error bars indicate a standard deviation. *N* = 8,  *P < 0.05,  **P < 0.01.

The fibronectin-1 gene expression was not changed substantially by the treatment of bFGF (Figure 2(a)). On the other hand, an increase in the type-I collagen gene expression was significantly induced by the treatment with bFGF in both young and adult groups. In addition, the type-I collagen gene expression was significantly (*P* < 0.01) up-regulated by bFGF in the young group than in the adult group (Figure 2(b)). 

**Figure 3:**
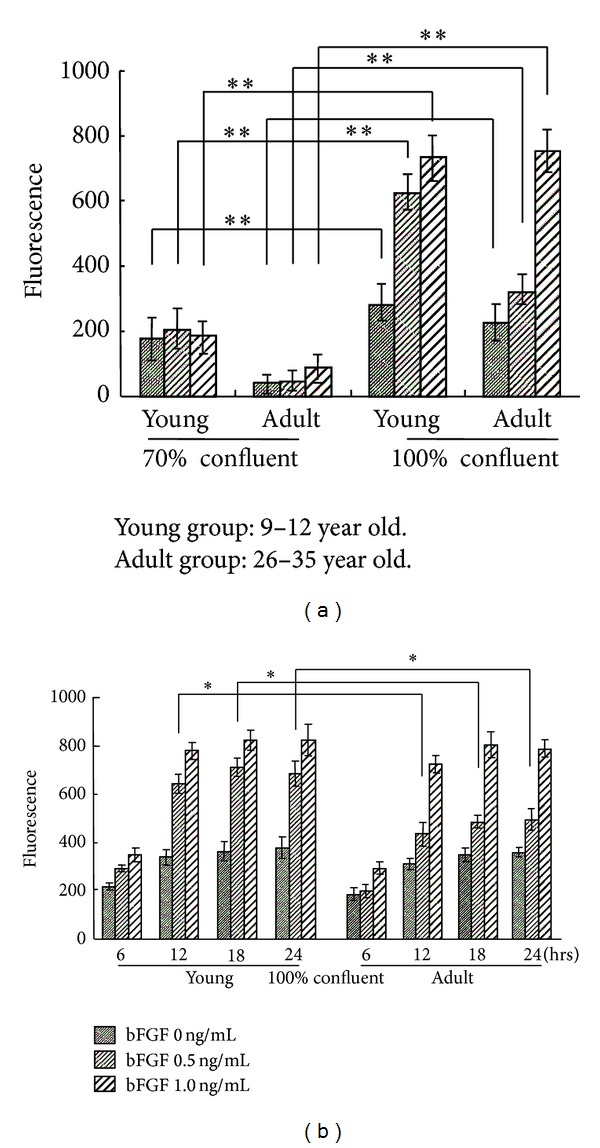
Effect of the activation of bFGF on the induction of apoptosis in cultured human GF. (a) Comparison of caspase-3 activity between 70% confluent and 100% confluent groups. (b) Time course of caspase-3 activity in 100% confluent group. Error bars indicate a standard deviation. *N* = 8,  *P < 0.05, **P < 0.01.

 The caspase-3 activity was enhanced significantly by the bFGF treatment for 12 hrs in human GFs at 100% confluence, while the bFGF treatment had no significant effect on caspase-3 activity in human GFs at 70% confluence (Figure 3(a)). Especially, 0.5 ng/mL bFGF enhanced the level of caspase-3 in GFs of the young group than that of the adult group; however when human GFs was treated with 1.0 ng/mL bFGF, the activity of caspase-3 was almost the same between young and the adult groups. (Figure 3(b)). Meanwhile, the caspase-3 activity in GFs at 100% confluence increased in a time-dependent manner and reached a plateau at 12 hrs (Figure 3(b)). 

**Figure 4:**
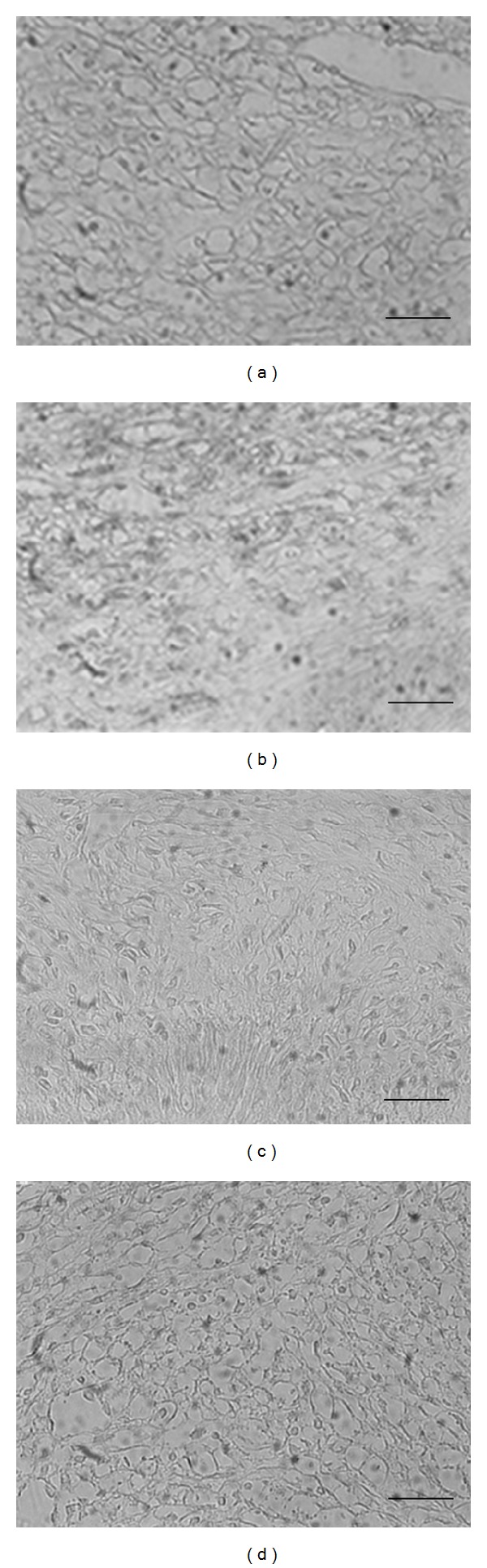
Effect of bFGF on apoptosis in rat palate. At 10 days after the excision, the head was resected under deep anesthesia with diethyl ether and fixed by immersion. Paraffin sections of the rat palate 6 ((a), (b)) and 12 weeks ((c), (d)) were stained with ApopTag Plus Peroxidase In Situ Apoptosis Detection Kit and observed by light microscopy. (a), (c): control group; (b), (d): bFGF injection group. The scale bars represent 25 *μ*m.

 In the injection group, more than 60% of the cells in 6 weeks Wistar rats had condensed nuclei and reduction of cytoplasm, which are typical of apoptosis (Figure 4(a)), whereas less than 10% of the cells had the features of apoptosis in the 12 weeks Wistar rats (Figure 4(b)).

## 4. Discussion

bFGF is widely known as a cellular growth factor and has a strong multiplication—stimulating activity for various kinds of cells such as osteoblasts, fibroblastic cells, and vascular endothelial cells [13]. It is documented that bFGF highly enhances the proliferation of immature human PDL cells while maintaining the differentiation potential and increases the size of stem cell clones in the human PDL [10, 14]. The injection of bFGF into skin wound sites reduced the degree of granulation tissue formation and increased apoptotic events [15]. We have shown that the injection of bFGF into rat palate wound sites increased apoptotic events. From these results, bFGF enhances the apoptosis of fibroblasts* in vivo* and inhibits the scar tissue formation.

In this study, bFGF was added to GFs until 80% confluence to examine the entire proliferation activity, whereas gene expression of the extra cellular matrix (ECM) was examined at 80% confluence. In the young group, a significantly higher proliferation activity was shown when compared to the adult group on days 5 and 7, while there were no differences between two the groups on day 9. Human GFs reached 80% confluent on day 9, and the cell proliferation decreased. The difference of proliferation of human GFs between two groups only appears during proliferation stage. On the other hand, bFGF treatment increased the proliferation of human GFs in both groups, and the days that reached the peak were shortened on day 5 from day 7 by the treatment of bFGF. In addition, gene expression of type-I collagen was increased by the addition of bFGF. In a previous study, when the difference in migratory competence was examined for fibroblasts from embryo and elderly subjects, the migratory capacity was lower in the adult cells than in the young origin cells [16]. Moreover, in experimental system to induce cell aging, a repeated subculture made a comparison between 10th and 20th passaged cells and demonstrated that rate of functional deterioration of migratory competence was 25% and 17% in 20th and 10th passaged cells [16]. It is thus shown that cell activity reduces with aging.

In addition, we investigated the apoptosis induction potency of bFGF. For the induction mechanisms of apoptosis, there are various apoptotic signal transduction pathways, in which the major one is through death receptors and mitochondria. Mitochondria plays an essential role in many forms of apoptosis by releasing apoptogenic factors such as cytochrome c [17] and apoptosis-inducing factor (AIF) [18] which activates death proteases called caspases [19]. Active effector caspases such as caspase-3, -6, and -7 mediate the cleavage of an overlapping set of protein substrates, resulting in morphological features of apoptosis. It is considered that apoptosis is surely induced for the detection of revitalization, because the examination of apoptosis in the actual experiment observes reaction of caspase-3 located in the final phase in the downstream. It is considered that apoptosis has been generated when the reaction of caspase-3 increases. 

In this study, to clarify the influence of host ages on the apoptosis induction potency of bFGF, the induction of caspase-3 by bFGF was examined. The reaction of caspase-3 in GFs with various cell densities was examined. When 70% confluent GFs was treated with bFGF, the caspase-3 activity was significantly less than that of 100% confluent GFs. Moreover, 0.5 ng/mL bFGF significantly enhanced the level of caspase-3 in GFs in the young group than in the adult group. However when human GFs was treated with 1.0 ng/mL bFGF, the activity of caspase-3 was almost the same between the young and adult groups. In the previous study, fibroblast growth factor receptor (FGFR)-1 gene expression decreased relevant to age [20]. It is thus suggested that the cell response to bFGF was less in adult group than in the young group, and this difference was related to the dose of bFGF.

It is reported that bFGF effectively induced caspase-3 activation and apoptosis in transforming growth factor (TGF)-*β*1-pretreated granulation tissue-derived fibroblasts (GF-1) [9]. In our study, bFGF treatment induces apoptosis in human GFs without pretreatment of TGF-*β*1, indicating bFGF has direct effect on apoptosis. 

On the other hand, our study showed that injection of bFGF into rat palate wound sites increased apoptotic events. It is reported that bFGF injection into wound site induced a rapid increase in TGF-*β*1 [9]. Thus, TGF-*β*1 and bFGF levels at the injury site lead the apoptosis in fibroblasts; this result may contribute to the proliferative granulation tissue formation.

It became clear that once the cells reached the confluence, the apoptotic activity was enhanced. The difference in the action of bFGF due to the cell density of fibroblasts is assumed to accelerate wound healing. That is, bFGF stimulates the fibroblasts with the physiologically active substance discharged from the white blood corpuscle and macrophage at the inflammation period. The tissue defect is filled with the granulation tissue, and then the number of proliferating cells is decreased by apoptosis at the restructuring period, resulting in excessive extracellular substratum [6, 21].

In conclusion, cell proliferation and matrix synthesis are modulated by exogenous bFGF in human GFs. Furthermore, it is suggested that apoptosis in human GFs and rat palate was increased by the treatment with bFGF. From these findings, apoptosis induced by bFGF in fibroblasts may lead to granulation tissue formation and scar-less repair, moreover this effect of bFGF may be related to the host age.

## Figures and Tables

**Table 1:** Sequences of PCR primer.

Gene	Primer sequences
Type-I collagen	Forward 5′-CAAGAACCCCAAGGACAAGAA-3′
	Reverse 5′-CTTGCAGTCGTAGGTGATGTT-3′
Fibronectin-1	# 4450183 (search LC, Heiderberg)
GAPDH	Forward 5′-ATCATCCCTGCATCCACT-3′
	Reverse 5′-GTCATCATACTTGGCAGGTTTC-3′
